# Diagnostic Methods of *Clostridioides difficile* Infection and *Clostridioides difficile* Ribotypes in Studied Sample

**DOI:** 10.3390/antibiotics10091035

**Published:** 2021-08-25

**Authors:** Elena Novakova, Zuzana Stofkova, Vladimira Sadlonova, Lukas Hleba

**Affiliations:** 1 Jessenius Faculty of Medicine in Martin, Comenius University in Bratislava, Mala Hora 4A, 03601 Martin, Slovakia; elena.novakova@uniba.sk (E.N.); vladimira.sadlonova@uniba.sk (V.S.); 2 Faculty of Biotechnology and Food Sciences, Slovak University of Agriculture in Nitra, A. Hlinku 610/4, 94901 Nitra, Slovakia; lukas.hleba@gmail.com

**Keywords:** *Clostridioides difficile* infection, multi-step algorithm, multiplex “real-time” PCR, PCR capillary-based electrophoresis ribotyping

## Abstract

Background: *Clostridioides* (*Clostridium*) *difficile* is the most common nosocomial pathogen and antibiotic-related diarrhea in health-care facilities. Over the last few years, there was an increase in the incidence rate of *C. difficile* infection cases in Slovakia. In this study, the phenotypic (toxigenicity, antimicrobial susceptibility) and genotypic (PCR ribotypes, genes for binary toxins) patterns of *C. difficile* isolates from patients with CDI were analyzed, from July to August 2016, taken from hospitals in the Horne Povazie region of northern Slovakia. The aim of the study was also to identify hypervirulent strains (e.g., the presence of RT027 or RT176). Methods: The retrospective analysis of biological samples suspected of CDI were analyzed by GDH, anaerobic culture, enzyme immunoassay on toxins A/B, multiplex “real-time” PCR and PCR capillary-based electrophoresis ribotyping, and by MALDI TOF MS. Results: *C. difficile* isolates (*n* = 44) were identified by PCR ribotyping, which revealed five different ribotypes (RT001, 011, 017, 081, 176). The presence of hypervirulent RT027 was not identified. The *C. difficile* isolates (RT001, 011, 081, 176) were susceptible to metronidazole and vancomycin. One isolate RT017 had reduced susceptibility to vancomycin. A statistically significant difference between the most prevalent PCR ribotypes, RT001 and RT176, regarding variables such as albumin, CRP, creatinine, the length of hospitalization (*p* = 0.175), and glomerular filtration (*p* = 0.05) was not found. Conclusion: The results of PCR capillary-based electrophoresis ribotyping in the studied samples showed a high prevalence of RT176 and 001.

## 1. Introduction

*Clostridioides* (*Clostridium*) *difficile* is the most common nosocomial pathogen and antibiotic-related diarrhea. It causes and poses a significant medical and economic burden in healthcare facilities [1]. According to the European Centre for Disease Control and Prevention’s (ECDC) point prevalence survey of healthcare-associated infections (HAI), C. difficile was the eighth most frequently found microorganism [2]. In Europe, over the last two decades, there has been an increase in the incidence of *Clostridioides difficile* infection (CDI) cases and severity of CDI infections, and new highly virulent *C. difficile* strains (e.g., RT027) and other hypervirulent strains have emerged. [1] The ECDC started coordinating the surveillance of CDI in EU countries in 2016 [3]. The overall mean CDI density was 2.8 (95% CI 1.8–3.9) cases per 10,000 patients/days and community-associated CDI (CA-CDI) with an incidence density of 0.4 (95% CI 0.2–0.6) cases per 10,000 patients/days [4]. From 2010 to 2017, the incidence of CDI in Slovakia increased from 0.9 to 20.6/10,000 hospitalized patients [5]. The diagnosis of CDI is based on clinical symptoms accompanied by microbiological evidence of toxins produced by *C. difficile* or toxigenic strains of *C. difficile* [6]. The disease is multifactorial and environmental factors seem to set the conditions for *C. difficile* development [7].

The accurate and fast diagnosis of CDI is essential for optimal patient care and preventing the spread of infection [8]. Diagnostic methods for the identification of different targets determine the presence of free toxins or toxigenic strains in the diarrheal feces. The methods that detect the presence of *C. difficile* include evidence of glutamate dehydrogenase enzyme (GDH) and anaerobic culture, and methods that detect the presence of a toxigenic *C. difficile* [6]. The importance of the real-time PCR (polymerase chain reaction) method implementation lies in the high sensitivity and specificity of the testing method. The assay has high negative predictive value (NPV) and, therefore, can be used to accelerate the exclusion of *C. difficile* infection. The PCR can help early diagnostics and the early recognition of patients with *C. difficile* before complications occur [9]. The European Society of Clinical Microbiology and Infectious Diseases (ESCMID) recommends a multi-step algorithm for the CDI diagnosis [10]. ESCMID’s recommendations about multistep algorithm testing describe how a positive first test should be confirmed with one or two confirmatory tests—glutamate dehydrogenase enzyme (GDH), toxins A and B, or a polymerase chain reaction (PCR test) [11]. The controversy remains about the need to treat patients with evidence of *C. difficile* and negative toxin A/B enzyme immunoassay (EIA), as they might have undetectable toxin levels or asymptomatic colonization with *C. difficile* [12]. Hypervirulent RT027, which is also characterized by the high production of toxins A and B, is a more severe disease course with more frequent recurrences, and higher morbidity and mortality have been reported in relation with this strain. Further analyses have shown that there are more ribotypes with similar properties [13] as the RT176.

The strain BI/NAP1/027 contains a nucleotide mutation at position 117 on the *tcdC* gene that encodes the protein C, which causes the suppression of genes for A/B toxins. In addition, RT027 produces so-called binary toxins [14].

**Figure 1:**
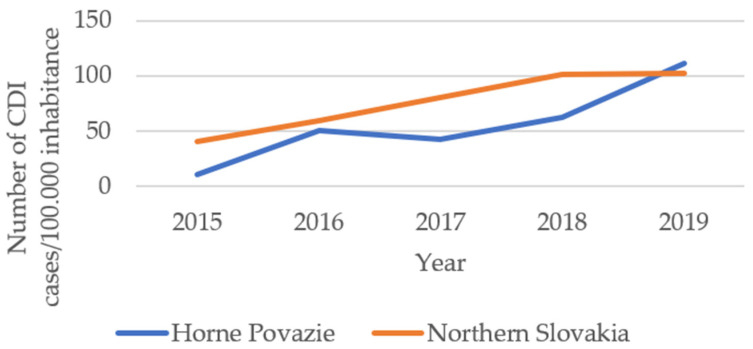
Number of CDI cases/100,000 inhabitants. Source: own processing according EPIS, 2020.

The purpose of the paper was also to identify the presence of hypervirulent strains, such as RT176 and RT027. The RT027 strain is referred to as *C. difficile* BI/NAP1/027 in the studied sample and the circulation of ribotypes among departments of in- and out-patients was monitored [15]. In a recent extensive study [16], it was shown that the effect of individual ribotypes on overall disease progression, mortality and biomarkers varied. In addition to *C. difficile* PCR ribotype 027, there are other strains that are associated with epidemics and a severe course of *C. difficile* infection. Despite the increased virulence of certain ribotypes, the PCR ribotype value as a predictor of disease severity is limited because the ribotype involved in the infection is not known until it is diagnosed. However, in epidemics, the ribotype could be considered when deciding on the choice of empirical treatment [10]. According to the data reported to the Epidemiological Intelligence Information System (EPIS) in the Horne Povazie region of northern Slovakia, the incidence of reported CDI cases had increased. In 2015, there were four patients/10,000 population reported. In 2017, the incidences doubled and, in 2018, the incidences increased up to 10 per 10,000 inhabitants. In 2019, this number remained approximately on the same level with 10.2 CDI patients per 10,000 inhabitants. About 84% of CDI patients were hospital-associated infections (HAI-CDI), and 16% of CDI patients were community associated (CA-CDI). In 2019, HAI-CDI cases from internal medicine, long-term care, intensive care unit, surgery, infectious diseases, and other wards were reported in Slovakia in 2019 (in a population of 100,000)—Figure 1. This increased incidence rate lies also in the higher testing rate and conscientious reporting to the Epidemiological Information System (EPIS) since 2016.

## 2. Results

Sixty *C. difficile* isolates included in the studied samples were tested for the presence of toxin genes by multiplex real-time PCR assay. [17] Twenty-eight isolates were from males and thirty-two isolates were from females. The median age was 77 years. The *C. difficile* isolates were taken from patients from the internal medicine (29 samples), long-term care (26), and surgery (1) wards, and from non-hospitalized patients (4). The average length of patient hospitalization was 40 days.

**Figure 2:**
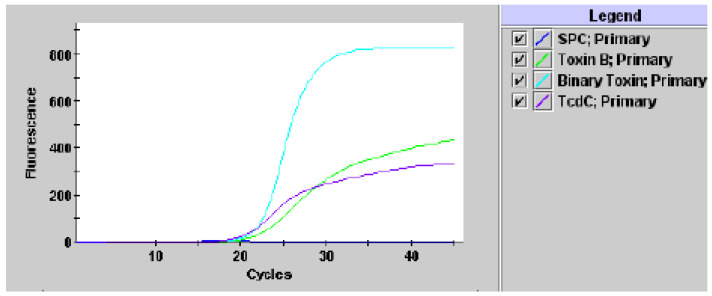
Positive result for toxigenic *C. difficile*, presumptive of 027/NAP1/BI [17].

Eighteen isolates (18) had positive results for the B toxin gene (*tcdB*), and genes for binary toxins (*cdtA* and *cdtB*) and the presence of nucleotide deletion 117 on the *tcdC* regulatory gene were negative. Forty-one (41) isolates carried both the B toxin gene and the binary toxin genes (*cdtA* and *cdtB*), as well as the presence of nucleotide deletion 117 on the *tcdC* regulatory gene for B toxin. Another investigated sample was negative for all investigated toxin genes—Figure 2.

**Figure 3:**
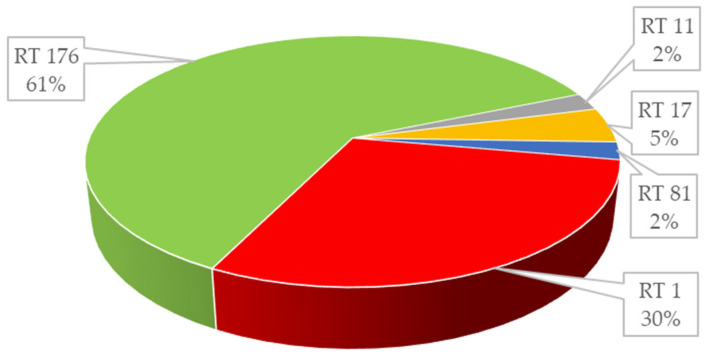
The proportion of ribotypes in *Clostridioides difficile* isolates (*n* = 44).

Due to the severe course of the disease, the *C. difficile* isolates were further analyzed by PCR capillary-based electrophoresis ribotyping. In the studied samples, five (5) different ribotypes of *C. difficile* isolates were revealed (RT001, 011, 017, 081, 176), which were identified by PCR ribotyping. The proportion of *C. difficile* in 44 isolates was: RT176 (*n* = 27; 69.5%), RT001 (*n* = 13; 23.7%), RT011 (*n* = 1; 1.7%), RT081 (*n* = 1; 1.7%), RT017 (*n* = 2; 3.39%)—Figure 3.

The PCR capillary-based electrophoresis ribotypes of *C. difficile* isolates on the strain level in patients (*n* = 44) were from internal medicine RT001 (6), RT176 (15), RT011 (1), RT081 (1); long-term care RT 001 (6), RT176 (12), RT017 (2); and surgery RT001 (1).

The most frequently occurring PCR ribotypes in the studied samples were RT176 and RT001. These ribotypes were present in patients’ samples from in-patient departments (internal medicine, long-term care ward) but also in outpatients that had a history of prior hospitalization.

**Figure 4:**
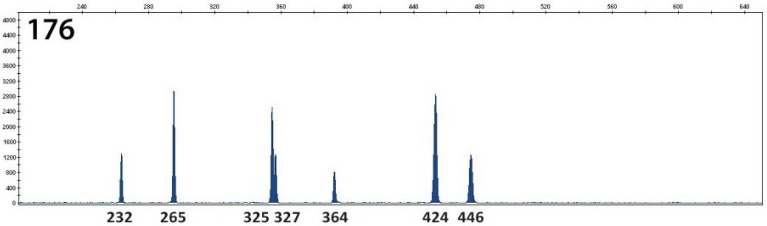
PCR capillary-based electrophoresis ribotyping on the strain level-RT176.

The presence of RT027 was not confirmed in any tested *C. difficile* isolate. The presence of RT176 (genetically very close to RT027) was confirmed in 27 samples—Figure 4. The importance of anaerobic cultivation resides in the determination of *C. difficile* susceptibility on antimicrobial agents. The *C. difficile* isolates (RT001, 011, 081, 176) were susceptible to metronidazole and vancomycin. One isolate RT017 had reduced susceptibility to vancomycin (Table 1).

The patients were typically hospitalized several times with chronic diseases, such as cardiovascular (29%), gastro-intestinal (24%), pulmonary (11%), renal (5%) and oncological diseases (1%), and other chronic diseases.

In the selected samples of patients, *C. difficile* isolates (*n* = 44) were analyzed following laboratory parameters, such as values of serum creatinine, total proteins, albumin, CRP, glomerular filtration and other parameters, such as the length of hospitalization. These parameters were compared between patients with ribotype RT001 and RT176. The dependent variable was the PCR ribotype and independent variables were the laboratory and other parameters such as the length of hospitalization.

The group infected with RT176 did not have a significantly different total protein level or level of albumin versus the group infected with RT001 (*p* = 0.300 and *p* = 0.682). Patients with RT001 had a lower level of glomerular filtration than those with RT176 (*p* = 0.054). There was no significant difference in the proportion of patients with an increase in serum creatinine concentration between RT001 and RT176.

Serum creatinine concentration data were also analyzed by gender. The different reference ranges were taken into account for the analyte in males and females. There was no significant difference in the level of CRP concentration between patients with RT176 isolates and RT001 isolates (*p* = 0.295).

There was a difference in the length of hospitalization (*p* = 0.175) between patients with RT001 and RT176. The mean duration of hospitalization for RT001 and RT176 patients was 44 and 35 days, respectively. Statistically significant dependence on identified ribotypes RT001 and RT176 were not found among the independent variables, serum creatinine (*p* = 0.524), total proteins (*p* = 0.300), albumin (*p* = 0.682), and CRP (*p* = 0.295) Table 2.

All laboratory variables were analyzed by gender as well. There were no differences between male and female patients in four studied variables. The all-cause mortality was 11/14 (79%) in patients with RT001 and 18/41 (67%) in patients with RT176, respectively.

## 3. Discussion

Multi-step diagnostic algorithms combining GDH and toxin EIA with PCR are recommended for CDI’s diagnosis [18].

The PCR tests are very sensitive to *C. difficile* but do not distinguish between symptomatic CDI and asymptomatic colonization, as they determine the genes for the production of toxins [9]. Genetic evidence of the toxigenic strain does not automatically mean that toxins are produced [19,20].

According to ESCMID, the use of one standalone CDI test is not recommended due to the low positive predictive value at low CDI prevalence [8].

The PCR ribotyping is essential for monitoring the spread of CDI and the course of the disease, as well as the detection of resistance to antimicrobial agents. This method is performed to identify individual strains, and to carry out surveillance of CDI spreading. According to the largest pan-European study of *C. difficile,* closER (2011–2015), 264 distinct ribotypes were revealed. The diversity between ribotypes varied markedly between countries and the years of the study. Epidemic and highly prevalent ribotypes included:RT027—prevalence 11.04%, RT014—9.1%, RT001—8.0%, RT078—6.5%, RT017—1.7%, RT176—1.3%, RT 011—1.1%, RT081—1.1%, and other ribotypes. The predominance of RT001 was reported in 2011 in Slovakia. The higher RT diversity was identified in 2012 (RT001, 014, 017, 081 and other ribotypes). This indicated lower antimicrobial resistance levels in countries with a greater *C. difficile* RT diversity [21]. In previous CDI studies, RT001 was also identified as predominant in Slovakia [22,23]. The new epidemic strains are less sensitive to antibiotics, e.g., resistant to fluoroquinolones. For many *C. difficile* strains, VAN susceptibility decreases gradually, which can be demonstrated by the increasing MIC. Reduced MTZ susceptibility was mainly observed in RT027 and RT198, and VAN resistance was observed in RT018 [21]. The occurrence of this ribotype 027/NAP1/BI *C. difficile* was also identified in countries in Central Europe [24]. The epidemic ribotypes exhibited a high level of antimicrobial resistance (RT017, 018 a 356) [25]. According to a study carried out in Slovakia, 2016, the occurrence of RT001 (59%), RT176 (23%), and RT027 (in 1 isolate) was found in 78 isolates [4]. The incidence of RT176 was also found in the Czech Republic [26]. The reduced susceptibility to moxifloxacin was identified in RT001, 017, 027, 176, in studies carried out by Krutova et al. and Freemann et al. [21,27].

Due to the epidemic situation during the period of our studied sample collection, there was a higher proportion of RT176 than RT001 identified. There was no RT027 identified in the studied sample. RT176 is close to ribotype RT027 and can be misidentified by commercial assays aimed at the deletion of one base pair at nucleotide 117 in the *C. difficile tcdC* gene that causes the suppression of genes for A and B toxins. The RT176 is also associated with a more severe course of the disease. RT176 is suggested to be related to RT027, since they belong to the same multilocus sequence [28].

Antimicrobial susceptibility testing investigated susceptibility to metronidazole in all *C. difficile* isolates. There was no confirmed reduced susceptibility or resistance to vancomycin in *C. difficile* isolates. Only one *C. difficile* isolate (RT017) had reduced susceptibility to vancomycin.

The link between clinical courses and specific ribotypes is still being investigated [29]. A hypervirulent RT027 is known worldwide. Some studies point to a more severe course of CDI disease when this ribotype occurs. The hypervirulent strain is referred to as *C. difficile* BI/NAP1/027 [15].

Some study results have supported that there may be other attributes of the *C. difficile* genome. These can significantly affect virulence (not only binary toxins and *tcdC* deletion), and hence the clinical course of the disease, which should be taken into account in the treatment strategy management [30]. In our studied sample, the RT027 was identified.

## 4. Materials and Methods

### 4.1. Sampling

The *C. difficile* strains isolated from 60 patients were analyzed in the laboratory in Klinicka Biochemia, Inc., Zilina, Slovakia which received fecal samples of patients suspected of CDI from 2 hospitals in northern Slovakia (with 1250 patient beds) and outpatients from a region which is inhabited by 251,202 citizens. The study investigated the prevalence of genotypic features (PCR ribotypes, genes for toxins A and B, and binary toxins), and phenotypic (toxigenicity, antimicrobial susceptibility) patterns of *C. difficile* isolates from a region of northern Slovakia were analyzed retrospectively from July to August 2016, by following multiple laboratory methods. There was no post-discharge follow up regarding readmission of the patients.

### 4.2. Enzymatic and Immunoenzymatic Assays and Cultivation

The samples were tested by direct diagnostic methods for *C. difficile* using the detection of GDH by the immunochromatographic method for toxins A or B (CERTEST *C. difficile* Toxin A/B), enzyme immunoassay methods ELISA (ProSpectT *C. difficile* Toxin A/B Microplate assay) to determine toxins A and B. The samples were mixed with an equal volume of 100% ethyl alcohol for 60 min at room temperature. One hundred microliters of the mixture was inoculated into *C. difficile* selective media (cycloserine-cefoxitin-fructose agar) (Brazier, Oxoid, Hampshire, UK). The plates were incubated at 37 °C for 72 h under anaerobic conditions [31].

### 4.3. MALDI-TOF MS Identification

The colonies of *C. difficile* were identified at the species level by Matrix Assisted Laser Desorption/Ionization Time-of-Flight Mass Spectrometry (MALDI-TOF MS), with the use of MALDI Biotyper v 3.0 system (Brucker Daltonics, Billerica, MA, USA). Intact proteins were isolated by the standard procedure with ethanol/formic acid/acetonitril extraction. *C. difficile* samples were overlaid with 1 µL of a matrix solution (HCCA = α-cyano-4-hydroxy-cinnamic acid saturated in solution containing 50% acetonitrile, 47.5% ultra-pure distillated water, and 2.5% trifluoracetic acid). Samples were dried at room temperature.

### 4.4. Antibiotic Susceptibility Testing

The clinical *C. difficile* isolates were also analyzed by E-tests (Oxoid) with a defined Minimal Inhibitory Concentration (MIC) for vancomycin (VAN) and metronidazole (MTZ). The Minimal Inhibitory Concentration breakpoints for MTZ (2 mg/L), VAN (2 mg/L) were applied and evaluated according to the European Committee on Antimicrobial Susceptibility Testing (EUCAST) [25,32].

### 4.5. Polymerase Chain Reaction PCR Ribotyping

The isolated *C. difficile* strains were analyzed also by the multiplex “real-time” PCR - GeneXpert *C. difficile*/Epi PCR assay (Cepheid, Sunnyvale, CA, USA) for the detection of the B toxin gene (*tcdB*), the binary toxin genes (*cdtA* and *cdt B*), and the deletion of the *tcdC* gene on nucleotide 117(∆117), which allowed the presumptive identification of 027/NAP1/BI with reported sensitivities and specificities of 96.6% to 99.7% and 93.0% to 98.6%, respectively [17]. The strains were only isolated from diarrheal stool samples from patients with a severe course of the disease and were analyzed by PCR capillary-based electrophoresis ribotyping.

The PCR capillary-based electrophoresis ribotyping was performed according to the protocol [32]. In the studied isolates, multi-locus sequence typing using seven housekeeping genes (MLST) was also carried out [26]. The molecular typing of *C. difficile* was carried out in a specialized reference laboratory. The PCR capillary-based electrophoresis ribotyping patterns of investigated ribotypes and fragment analysis were performed by a DNA analyzer and by Gene Mapper v5.0 which were kindly provided by the Department of Microbiology of 2nd Faculty of Medicine of Charles University, and Motol University Hospital in Prague. This method is performed in CDI epidemics to identify individual strains, and to perform surveillance for the spread of CDI [33,34].

The samples were collected from patients in internal wards and wards for long-term care (wards from the higher occurrence of CDI). The stool samples were sent to the laboratory according to the criteria for testing (Bristol scale 5–7). The selected patient samples were analyzed for *C. difficile* isolates (*n* = 44) following laboratory parameters, such as values of serum creatinine, albumin, CRP, and the length of hospitalization. Laboratory parameters were compared between patients´ samples with ribotype RT001 and RT176.

### 4.6. Statistical Analysis

Group statistics were used to describe the basic features of the data. Independent sample tests, including the Leven´s test for Equality of Variances and the t-test for Equality of means, were used. The Pearson correlation method was used to determine the dependency between two variables and statistical significance was set as *p* < 0.001. Descriptive statistical tools, as well as frequency of infection by frequency histograms, statistical programs were utilized.

## 5. Conclusions

Accurate and fast diagnostics of CDI are essential for optimal patient care and preventing the spread of infection. Laboratory diagnosis involving a high-sensitivity screening assay, followed by a high specificity assay, is important for the treatment and diagnosis of the disease. This study investigates the prevalence, genotypic features (PCR ribotypes, genes for toxins A and B, and binary toxins) and phenotypic (toxigenicity, antimicrobial susceptibility) patterns of *C. difficile* isolated from patients with confirmed CDI. We identified the presumptive identification of 027/NAP1/BI in stool specimens, which contained toxigenic *C. difficile* but not 027/NAP1/BI. *C. difficile* isolates were investigated by PCR ribotyping, which identified five different ribotypes in forty-four isolates (RT176, 001, 011, 017, 081). The presence of the RT176 was confirmed in 27 isolates. In our studied samples, there was a higher proportion of RT176 identified than RT001 due to the epidemic situation in this period. The *C. difficile* isolates (ribotypes 001, 011, 081, 176) were susceptible to MTZ and VAN. One isolate RT017 had reduced susceptibility to VAN. Despite the limitation of the study, it highlights the prevalence of *C. difficile* RT176 in the epidemic situation during the studied period. A further study is needed to help clarify the interaction between ribotypes and other predictors and laboratory parameters.

## Figures and Tables

**Table 1:** Genotypic and phenotypic characteristics of *Cĺostridioides difficile* in the studied sample.

Number of *C. difficile* Isolates	Fenotype Characteristics of *C. difficile* *isolates*				Genotype Characteristics of *C. difficile* *isolates*			
	GDH/ Culture	Toxins A/B (Rapid Test, ELISA)	MIC_90_ µg/mL MTZ	MIC_90_ µg/mL VAN	Gene for B Toxin (*tcdB*)	Genes *cdtA, cdtB* for Binary Toxins	Deletion of nt 117 in *tcdC* Gene (Susp. RT 027)	RT Ribotypes
13	positive	positive	0.047	1.5	positive	Negative	Negative	001
27	positive	positive	0.5	1.0	positive	Positive	Positive	176
1	positive	positive	0.125	0.5	positive	Negative	Negative	011
2	positive	positive	0.125	2.0	positive	Negative	Negative	017
1	positive	positive	0.125	0.5	positive	Negative	Negative	081

**Table 2:** *Clostridioides difficile* isolates RT001 and RT176 and selected laboratory parameters in the studied samples.

Laboratory and Other Variables	Ribotypes	Mean (Median) Years	95% Confidence Interval for Mean	Pearson *p* < 0.001
Total proteins g/L	001	61.10 (60.0)	(56.1–66.1)	0.300
	176	57.27 (59.0)	(51.2–63.0)	
Albumin g/L	001	29.39 (28.95)	(27.56–31.21)	0.682
	176	28.65 (28.95)	(24.94–32.36)	
CRP mg/L	001	78.07 (82.0)	(54.80–101.32)	0.295
	176	56.75 (26)	(18.36–95.14)	
Glomerular filtration mL/s	001	0.861 (0.73)	(0.687–1.034)	0.054
	176	1.156 (1.32)	(0.875–1.436)	
Length of hospitalization days	001	43.78 (44.0)	(36.04–51.52)	0.175
	176	35.10 (30.5)	(23.72–46.47)	
Creatinine μmol/L	001	122.21 (112.0)	(90.6–153.7)	0.524
	176	106.85 (76.0)	(66.9–146.8)	

## Data Availability

Data are available from the corresponding author upon reasonable request.
